# Vitamin D: A Potential Mitigation Tool for the Endemic Stage of the COVID-19 Pandemic?

**DOI:** 10.3389/fpubh.2022.888168

**Published:** 2022-06-10

**Authors:** Daniela Briceno Noriega, Huub F. J. Savelkoul

**Affiliations:** Cell Biology and Immunology Group, Wageningen University & Research, Wageningen, Netherlands

**Keywords:** vitamin D, COVID-19, SARS-CoV-2, vitamin D supplementation, endemic, vitamin D deficiency, immunomodulation

## Abstract

The impact of the severe acute respiratory syndrome coronavirus 2 (SARS-CoV-2) pandemic and associated development of clinical symptoms of COVID-19 have presented an enormous global impact on our health care systems, public health and economy. To date several observational epidemiological studies consistently found that vitamin D deficiency, measured as low levels of circulating 25-hydroxyvitamin D, is associated with cardiovascular diseases, diabetes, certain cancers, autoimmune diseases and many infectious diseases, including acute respiratory infections. Since vitamin D is not merely immunosuppressive but also acts as an immunomodulator in tolerance and homeostasis, many experts have considered a role of vitamin D in the prevalence and severity of immune mediated inflammatory diseases, such as SARS-CoV-2, adding to the evidence of the importance of vitamin D in the immune response against viral respiratory infections and reinforcing the need for targeted vitamin D supplementation, with a focus on high-risk populations and a high-dose supplementation treatment for COVID-19 hospitalized patients. The expected transition to endemicity of SARS-CoV-2 even further corroborates as a potential of vitamin D as an potential mitigation tool for the prevention of COVID-19. The aim of this paper is to analyse the current evidence regarding vitamin D and present a hypothesis of its potential role in the current COVID-19 pandemic and in the future as a potential preventive measurement in public health.

## Introduction: Current Status Of The Covid-19 Pandemic

The new year begun with an overall consensus from the scientific community that coronavirus disease 2019 (COVID-19) will, at some point, become endemic. However, it is clear that in the first months of 2022 we are far away from that point in a pandemic that has lasted for more than 2 years. Presently, a percentage of the population still lacks any immunity, either from vaccination or natural infection plus with new variants consistently emerging, reaching the endemic stage still seems a long way away (1). Nonetheless, as we enter the third pandemic year, we face a new challenge. Now we need to determine what is the best way to manage the transition to endemicity using the tools we have at our disposal to mitigate the effects of severe acute respiratory syndrome coronavirus 2 (SARS-CoV-2) as it continues to circulate in our population (2). Endemicity has always been our best hope; eradication was an extremely high bar, considering that SARS-CoV-2 is a zoonotic disease; hence it has non-human reservoirs of infection. So far no zoonotic infectious disease has ever been eradicated. To date, only two diseases have ever been eradicated: smallpox and rinderpest; and unfortunately cases of polio still occur despite our best efforts (2, 3).

Vaccines have been highly effective in the fight against SARS-CoV-2, they protect against serious illness, hospitalization and death; additionally a third dose has shown to be is more effective in neutralizing the Omicron variant compared to a 2-dose mRNA vaccine regimen, though how long the immunity will last is yet unknown (4). Nonetheless, neither vaccination nor natural infection appear to provide lifelong immunity and vaccinating billions of people at regular intervals is an unrealistic goal (3). Moreover, even fully vaccinated individuals can experience a break-through SARS-CoV-2 infection; thus the endemic stage of COVID-19 means learning to live with this virus and adopt appropriate risk thresholds control methods that reflect hospitalizations and death rates, not merely daily cases (3).

In order to reach this stage it is necessary to implement public health mitigation strategies along with continued vaccination campaigns that will have an impact on improving the “respiratory health” of our communities and decrease the prevalence of viral respiratory illnesses along with SARS-CoV-2 hospitalization and death rates.

This review will focus on the reported evidence that proposes that vitamin D can reduce viral viability and replication by inducing antimicrobial peptides (AMPs), as well as reducing the production of pro-inflammatory cytokines; among additional important immunomodulatory effects (5). Moreover, with respect to SARS-CoV-2, viral mutations can reduce the adaptive immune system's ability to respond effectively, which is not the case with the innate immune system response; therefore the interaction between vitamin D and the innate immune system is not sensitive to the emerging variants of SARS-CoV-2 (6). Hence, vitamin D will remain an effective immunomodulator regardless of the upcoming variants in the COVID-19 pandemic, including the period of endemicity. Thus, the role of vitamin D as an immunomodulator and the reported effects of vitamin D supplementation and COVID-19 severity (7) make vitamin D an interesting potential mitigation tool for the endemic stage that can impact the hospitalization rates as we learn to live with COVID-19. The present review will explore the role of vitamin D as an immunomodulator and the reported evidence during the COVID-19 pandemic of the role vitamin D deficiency and supplementation can play in SARS-CoV-2. Finally, the possible role vitamin D supplementation can play as the COVID-19 pandemic enters the endemic stage will be discussed.

## Vitamin D

Even in pre-pandemic times, vitamin D supplementation has proven to be a controversial subject. It has been suggested for several years now that low levels of vitamin D increase the risk for developing disease (8–13), although this claim has been debated (14); with randomized control trials (RCTs) and a few meta-analyses not been designed to yield definitive conclusions (15). When assessing vitamin D status, serum circulating 25-hydroxyvitamin D [25(OH)D] is currently the main plus the preferred indicator of vitamin D status, it can be reported in both nmol/L or ng/mL (15). An important caveat is that serum levels of 25(OH)D may not reflect tissue levels of active vitamin D (16). It is important to note that occasionally, the term “vitamin D” can be used interchangeably for the different forms vitamin D (as shown in Table 1). For the purpose of this review, when the term vitamin D is used, unless noted otherwise, we are referring to 25(OH)D.

**Table 1:** Vitamin D [25 (OH)D] range guidelines from various organizations, equivalences and terminology.

**Serum Levels [25 (OH) D] ng/mL**			
	**Vitamin D**	**Endocrine**	**Food and nutrition**
	council*	society**	board***
Sufficient	40–80 ng/mL	30–100 ng/mL	>20ng/mL
Insufficient	31–39 ng/mL	21–29 ng/mL	12–20 ng/mL
Deficient	0–30 ng/mL	0–20 ng/mL	0–11 ng/mL
**Vitamin D equivalences**			
1 μg = 2.5 nmol			
1 μg = 40 IU			
1 ng/mL = 2.5 nmol/L			
**Terminology**			
25-hydroxyvitamin D = [25(OH)D] = calcidiol			
1,25-dihydroxyvitamin D = [1,25(OH)_2_D] = calcitriol			
Vitamin D = calciferol			
Vitamin D2 = ergocalciferol			
Vitamin D3 = cholecalciferol			

^*^Vitamin D Council: www.icwb.com/daily-vitamin-d-dosage-and-requirement. ^**^Endocrine Society: Holick et al. (17) “Evaluation, Treatment and Prevention of Vitamin D Deficiency” www.endocrine.org/clinical-practice-guidelines/vitamin-d-deficiency. ^***^Food and Nutrition Board: www.ods.od.nih/gov/factsheets/VitaminD-HealthProfessional/. (same values as the Institute of Medicine -IOM).

**Figure 1:**
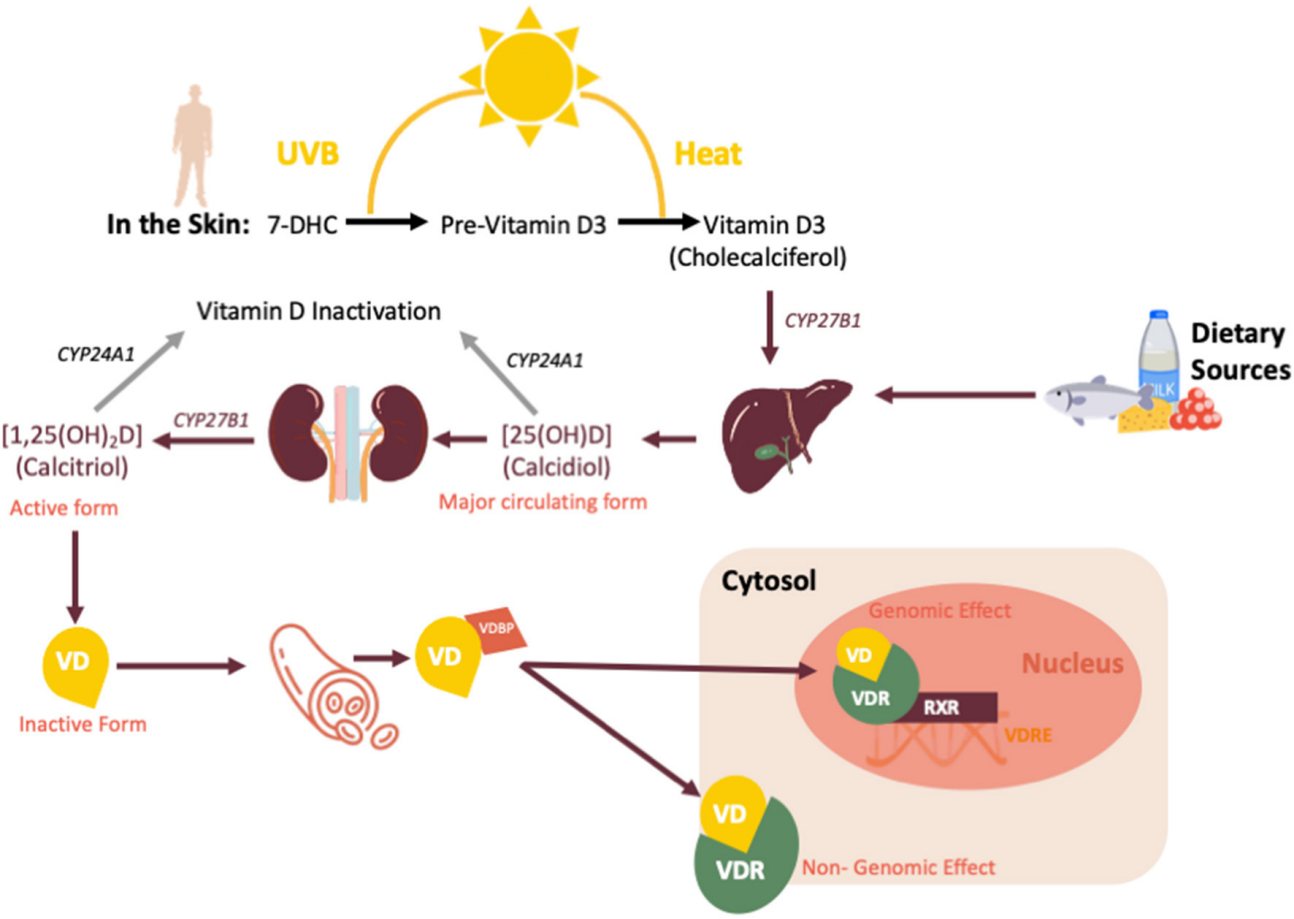
Vitamin D sources, synthesis and metabolism. CYP27B1, cytochrome P450 Family 27 Subfamily B Member 1; CYP24A1, Cytochrome P450 Family 24 Subfamily A Member 1; VD, vitamin D; VDBP, vitamin D binding protein; VDR, vitamin D receptor; VDRE, vitamin D response element; RXR, retinoid-X receptor.

Vitamin D is a fat-soluble hormone primarily synthesized in the skin during exposure to UVB radiation (as shown in Figure 1). The main steps in vitamin D metabolism are all performed by cytochrome P450 mixed-function oxidases (CYPs), including CYP27B1, which is also expressed in immune cells (18, 19). Cellular actions of the biologically active form of vitamin D [1,25(OH)_2_D or calcitriol], are mediated by the vitamin D receptor (VDR), a ligand-dependent transcription factor that regulates the expression of more than 900 genes involved in an array of physiological functions (20). Vitamin D plays a crucial role in calcium homeostasis; however, recently the numerous extra-skeletal actions of vitamin D have been elucidated, including important immunomodulatory properties (21). As an example, many observational studies have reported a protective role of vitamin D against tuberculosis, otitis media, bronchiolitis and viral wheezing (22, 23). Dogan et al. reported of a possible correlation between vitamin D deficiency (defined as a serum concentration of 25(OH)D ≤ 20 ng/mL or <50 nmol/L) and respiratory distress syndrome (RDS) in premature infants (24). The authors proposed a possible role of vitamin D in promoting lung maturity, because 25(OH)D could prevent surfactant insufficiency by increasing proliferation if type II pneumocytes (24).

Additionally, studies have argued that levels of vitamin D may contribute to immunoregulatory functions during viral respiratory infections by downregulating excessive cytokine responses and at the same time improving clearance of various microbial species. Several studies have observed an inverse association between vitamin D levels and incidence of a number of infections, including: influenza, upper respiratory tract infections, HIV infection and bacterial vaginosis (as shown in Supplementary Table 1) (25–32). The potential beneficial effects of vitamin D have been observed also in other infectious diseases, including hospital acquired *Clostridium difficile* infection (CDI) (33), recently in COVID-19 infection (34) and sepsis (35). Overall, there is supporting evidence that vitamin D has potential in preventing and reducing the severity, and possibly the complications, of several infectious diseases.

### Vitamin D as an Immunomodulator

**Figure 2:**
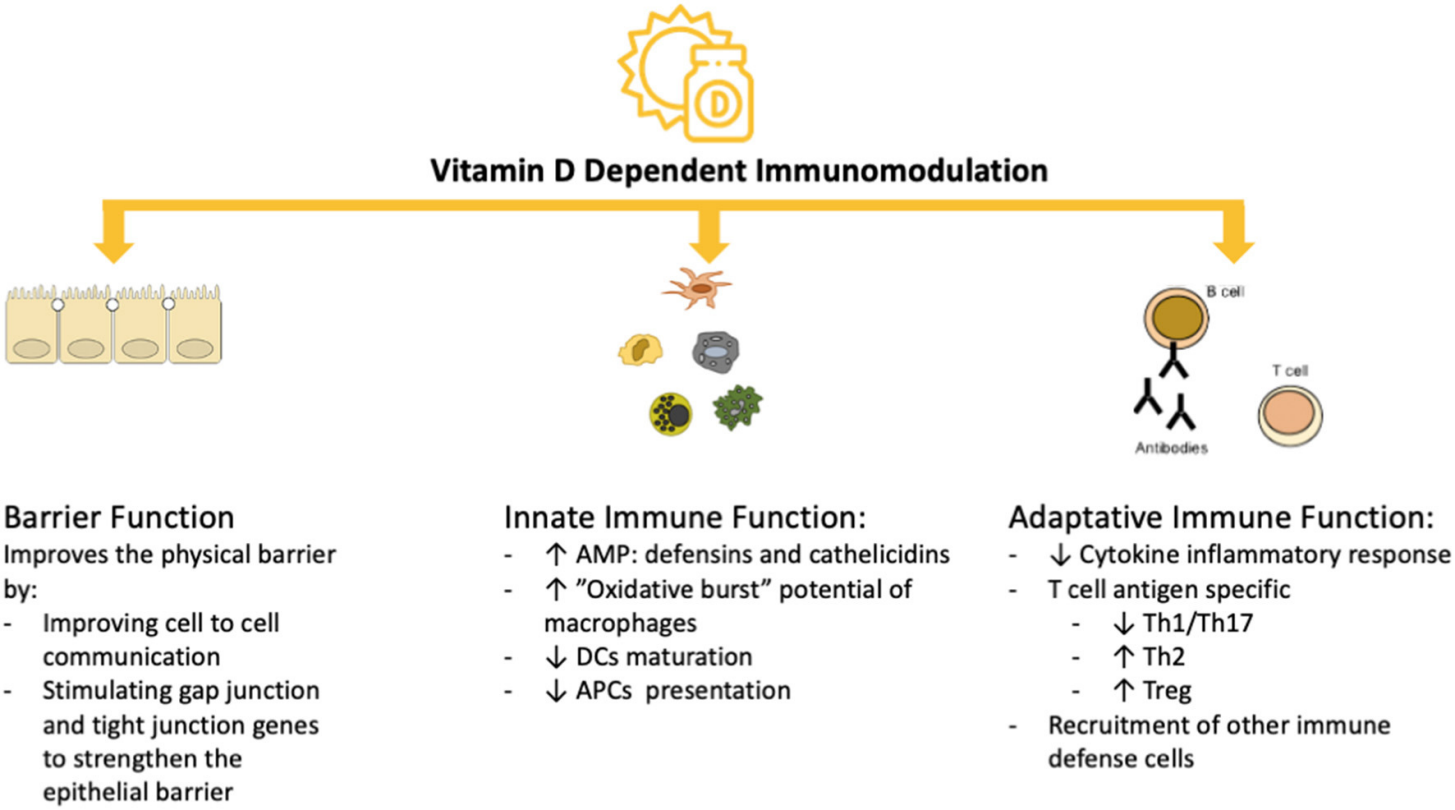
Vitamin D-dependent Immunomodulation. AMP, antimicrobial peptides; APCs, antigen presenting cells; DC, dendritic cells; Th1, type 1 T helper cells; Th17, type 17 T helper cells; Treg, regulatory T cells.

Vitamin D exerts its immunomodulatory activity by affecting the function of cells of the innate and the adaptive immune system, including dendritic cells (DCs), macrophages, T cells and B cells (as shown in Figure 2). The actions of vitamin D on the immune system are extremely complex due, in part, to the vitamin D receptor (VDR), which is expressed in many immune cells (36–46). The VDR, a member of the nuclear steroid receptor family, is encoded by the VDR gene on chromosome 12 and modulates gene transcription (47). The VDR mediates its actions by first binding with its ligand [1α,25(OH)2D3], then forming a heterodimer with the retinoid X receptor (RXR). This heterodimer binds to the promoter-proximal VDR response element (VDRE), thereby initiating recruitment of nuclear proteins into the transcriptional complex and modulating gene expression (as shown in Supplementary Figure 1) (47). The VDR is found in tissues throughout the body, including immune cells, with effects on apoptosis and cell differentiation. The DNA-bound VDR/RXR heterodimers can also downregulate transcription; for example in T cell cytokine production. Polymorphisms of the VDR gene could have significant effects on immune regulation by altering the differentiation and proliferation responses (48–51). Therefore, it is hypothesized that the response to vitamin D supplementation could be modulated by genetic variants in the VDR gene, of which Apal (rs7975232), BsmI (rs1544410), TaqI (rs731236) and FokI (rs10735810) are the most widely studied (47). A recent meta-analysis concluded that the TaqI and FokI VDR polymorphisms could play a role in the modulation of the response to vitamin D supplementation because they were associated with a better response to supplementation (52). Moreover, the most common polymorphisms in the vitamin D binding protein (VDBP) gene (rs4588 and rs7041) may correlate with differences in vitamin D status in the serum (51). Because of the very short half-life of free serum vitamin D, it is stabilized and transported to target tissues by being bound to the VDBP. Additionally, several polymorphisms (Bsm-I, Taq-I, Apa-I and Fok-I) in the VDR gene were modestly associated with several vitamin D-related diseases (8–13, 53). Recently, VDR gene polymorphisms were described to be independently associated with COVID-19 severity and survival (54). However, larger sized clinical studies together with tissue vitamin D levels are needed to determine the impact of polymorphisms on COVID-19.

In the innate immune system, the interaction of vitamin D with the VDR can regulate inflammatory immune responses by enhancing the expression of β-defensin and cathelicidin from Toll-like receptors (TRL)-stimulated monocytes, while inducing a more tolerogenic phenotype in DCs (21). Vitamin D-treated DCs showed decreased expression of co-stimulatory molecules CD40, CD80 and CD86 and decreased secretion of interleukin (IL)-12, while also inhibiting cell maturation and downregulating the VDR expression; thereby limiting the capacity for antigen presentation to T-cells and subsequent Th1 and Th17 protective anti-viral adaptive immune responses. Vitamin D can induce proliferation plus production of IL-1β and IL-8, while inhibiting the expression of TLR2 and TLR4 and the release of pro-inflammatory cytokines like IL-6 (21). Surprisingly, these activities of vitamin D on the innate immune system interfere with crucial processes that mediate the viral entry and induction of a subsequent innate immune response to SARS-CoV-2. While the angiotensin converting enzyme-2 (ACE2) receptor is crucial for viral entry into cells, innate receptors like intracellular or endosomal pattern recognition receptors (PRRs) sense viral components, including the envelop protein by TLR2, the spike protein by TLR4, single-stranded RNA by TLR7/8, double-stranded DNA intermediates by TLR3, and cytosolic RNA by RIG-I-like receptors. Antiviral and anti-bacterial innate immune responses are both based on initial detection through specific PRRs and the subsequent engagement of PRRs activates signaling pathways culminating in activated transcription factors, such as interferon regulatory factors (IRF), activation protein (AP)-1 and nuclear factor kappa-light-chain-enhancer of activated B cells (NF-kB) family members, that are crucially involved in the induction of cytokines, type 1 interferons and other antiviral effectors. AMPs like the positively charged LL-37 and HBD-2 bind to negatively charged viral nucleic acids, which enhances endocytosis and TLR3, TLR7 and TLR9 interactions. This results in enhanced type I interferon responses, which was recently shown to be also active in SARS-CoV-2 infection (55). As a consequence, a strong type I interferon (IFN-α/β) response is initiated, which is counteracted by other interactions including ORF9b, non-structural proteins 1 and 13, and the nucleocapsid viral protein (56). Collectively, these findings provide a theoretical landscape in which vitamin D could repress SARS-CoV-2 infection and aberrant immune response resulting in severe COVID-19.

Different innate immune cells, including neutrophils, express VDR and thus vitamin D exposure can result in NET formation, while inhibiting systemic inflammation; thereby enhancing bacterial killing while inhibiting inflammation (54). Besides phagocytosis and reactive oxygen species (ROS) production, neutrophils are versatile cells that are regulated by type I IFN-α/β. Viral infections, including SARS-CoV-2 infection, may result in emergency granulopoiesis characterized by the presence of interferon-stimulated genes (ISG)-related neutrophils with increased expression of CD64 and alarmin-related S100A genes, plus the presence of both immature neutrophils and mature populations in the peripheral blood which can induce an immunosuppressive or a pro-inflammatory response (56–59).

As mentioned before, several studies have argued that levels of vitamin D may contribute to immunoregulatory functions during viral respiratory infections, by downregulating excessive cytokine responses and improving clearance of various microbial species (60, 61). Vitamin D was shown to suppress IFN-γ production and induce IL-10 production in human CD4+ T cells by inducing epigenetic changes resulting in increased available chromatin leading to the upregulation of key transcription factors like STAT3 and the STAT3 activator IL-6. VDR complexed with vitamin D binds to the promoter of the STAT3, IL-6 and IL-10 genes; this may be critical in converting pro-inflammatory Th1 cells to regulatory T cells (Tregs) and other subsets that resolve type 1 immunity, which includes severe COVID-19 (16). Vitamin D can thus regulate mature T cell responses by inhibiting Th1 and Th17 differentiation and increasing the amount of CD4+ CD25+ T regulatory cells, by inhibiting the production of IFN-γ, IL-2 and tumor necrosis factor-alpha (TNF-α); as well as IL-17 and IL-6 (21, 54); at the same time, the production of IL-4, IL-5, IL-9 and IL-13 is increased by vitamin D (54). Thus, the production of vitamin D by macrophages results in a shift from a pro-inflammatory state to a more tolerogenic state (54).

An important target for the immunomodulatory effects of vitamin D are DCs, who act as antigen presenting cells (APCs) and play a role in maintaining peripheral tolerance; thus preventing autoimmune damage from self-reactive T cells (60). Vitamin D is also able to inhibit proliferation of activated B cells, induce B cell apoptosis and inhibit the generation of plasma cells and the production of antibodies (62). Additionally, vitamin D can induce the production of AMPs such as cathelicidins and β-defensins from neutrophils, macrophages and from epithelial respiratory cells (63–67). Cathelicidins have direct antimicrobial activities against a spectrum of microbes, including viruses and are also capable of promoting cellular autophagy of infected cells, thereby providing protection against viral infections (68, 69). Vitamin D also enhances the antimicrobial activity of M1 type macrophages by increasing the TLR and CD14 expression (66), promoting the migration of DCs to lymphoid organs where they can present antigens to T cells (67) and increasing the activity of NADPH-dependent oxidase (70). Activated pro-inflammatory M1 type macrophages, in *in-vitro* cultures, were shown to express higher levels of CYP27B1 than M2 type macrophages, thus producing more calcitriol and T cells; which in turn will increase the vitamin D production even further by expressing CYP27B1 (71). Moreover, vitamin D can inhibit the production of pro-inflammatory cytokines, which in viral respiratory infections can be beneficial to the host by reducing the so-called “cytokine storm” (72, 73). Therefore, overall there is supporting evidence that adequate levels of serum vitamin D have the potential of preventing and reducing the severity, possibly even the complications of several infectious diseases, particularly viral respiratory infections. Thus, currently the proposed role of vitamin D is widely accepted; concurring that vitamin D is not merely immunosuppressive but an immunomodulator in tolerance and immune homeostasis.

Regarding COVID-19, vitamin D potentially mitigates the risk of disease severity by reducing the production of pro-inflammatory cytokines; thereby diminishing the cytokine storm based on a dysregulation of the innate immune system which results in the outpouring of pro-inflammatory cytokines and chemokines leading to an abnormal activation of the adaptative immune pathway (70). Moreover, vitamin D promotes the suppressive function of T-regs, the suppressive cells of the adaptative immune system, which are critical for regulating the innate and the effector responses (45). SARS-CoV-2 infection leads to the onset of acute respiratory distress (ARDS) syndrome (74, 75). Vitamin D can potentially modulate these pathophysiological aspects via the VDR, since the airway epithelium can display transcriptional induction of 1α-hydroxylase (CYP27B1) which results in bioactive [1,25(OH)_2_D] and the expression of intracellular VDR. Moreover, vitamin D production can be further boosted by the presence of pulmonary macrophages, which express CYP27B1 and VDR upon viral exposure (68). Additionally, vitamin D can induce immune tolerance and has the capacity of promoting the release of anti-inflammatory mediators, such as IL-10, IL-35 and transforming growth factor β (TGF-β), thereby mitigating the cytokine storm (70, 73).

### Lessons Learned Regarding Vitamin D in COVID-19 Times

A systematic review of 39 studies reported a significant association between [25(OH)D] serum level and SARS-CoV-2 infection (75). Moreover, adjusted studies that used the Cox survival method (HR: 7.67; 95% CI 3.92, 15.03, I2: 0.0%) showed a significant association between mortality and vitamin D deficiency (75). However, studies that evaluated the association between vitamin D deficiency and intensive care unit (ICU) admission, hospitalization, pulmonary complications and inflammation showed inconsistent results (75). Moreover, in a double-blind, placebo-controlled randomized trial in hospitalized patients with moderate to severe COVID-19, supplementation with vitamin D3 did not show significant differences compared to placebo in the percentages of patients who were admitted to the ICU, who required mechanical ventilation or who died during hospitalization (76). At the same time, a randomized controlled trial (RCT) reported highly significant reduction (*p* < 0.01) in inflammatory markers (TNF-α, C-reactive protein) after vitamin D therapy supplementation of 4,000 IUs of vitamin D3 at 3 times per week for 6 months in patients with type 2 diabetes (77). The proposed conclusion of these studies is that vitamin D supplementation appears to inhibit inflammatory markers also associated with COVID-19 without any side effects in hospitalized patients with mild to moderate symptoms, but it would be ineffective in severe cases. Moreover, after vitamin D therapy, serum levels increased within about 10 days from >20 ng/ml to 80–100 ng/ml, suggesting that it is more effective to give daily doses compared to a high bolus, and to provide enough time to obtain an elevation in the serum levels of vitamin D (77, 78). This difference in the effectiveness of daily or weekly doses vs. a single high bolus dose of vitamin D is a known recommendation. Martineau et al. reported a meta-analysis of individual participant data analyzing 25 RCTs and concluded from their analysis that vitamin D supplementation could prevent acute respiratory tract infections among all participants (adjusted odd ratios 0.81, 95% CI 0.81 to 0.96; P for heterogeneity < 0.001) (79). Crucially, in a subgroup analysis, the protective effects of vitamin D supplementation were stronger in those receiving daily or weekly doses and in participants with baseline 25(OH)D levels <25 nmol/L (79). Thus, this meta-analysis conducted in the pre-COVID-19 era, provides important lessons as to what is the most effective way to supplement patients with vitamin D in order to observe protective effects. Additionally, it has been reported that physiological levels of 30–50 ng/mL of 25(OH)D are sufficient to modulate inflammatory activities by inhibiting pro-inflammatory cytokine production (80, 81). This threshold needs to be considered when evaluating the effects of vitamin D supplementation, particularly during the COVID-19 pandemic in which vitamin D could play a protective role (82). The VITdAL-ICU RCT analyzed the effect of high dose vitamin D3 in critically ill patients and followed them for 6 months (83). At the 6 month follow-up lower mortality was observed among the vitamin D3 supplementation group (28.6%, 95% CI, 19.9–38.6%) compared to the placebo group (46.1%, 95% CI 36.2–56.2%); additionally significantly better hand grip strength and physical performance in the vitamin D3 group (83). These findings suggest a potential role for vitamin D supplementation in the recovery phase as well as during disease progression in SARS-CoV-2 infection (83).

An additional point to consider when discussing the effects of vitamin D is the role of calcium, since insufficient calcium intake and vitamin D deficiency are not only common but usually concurrent (84). However, it is essential to remember that they not only reflect different entities that play different roles but tissue production of vitamin D by cells expressing 1α-hydroxylase, as the product of the CYP27B1 gene, is regulated by calcium independent mechanisms (21). In coronavirus infections, calcium mediates the fusion of the viral envelope with the host cell membrane either by the viral spike fusion protein subunit S1/S2 or the transmembrane domain of the ACE_2_ receptor (84). This results in impaired conversion of angiotensin II (Ang-II) into angiotensin 1–7 and in the subsequent development of a cytokine storm as well as tissue damage. Vitamin D affects endothelial cell function, thereby regulating vasodilatation although direct proof of the causal role of endothelial dysfunction in the pathogenesis of vitamin D deficiency is still lacking (85). Together, these changes contribute to the development of ARDS (86). Hence, vitamin D would play a protective role in ARDS by inhibiting renin expression and the ACE/angiotensin II receptor type I (AT1R) axis plus stimulating the ACE2/Ang (1-7)/Mas G (G-protein-couple receptor Mas) (87).

## Vitamin D Deficiency

Currently there is no consensus regarding the optimal levels of vitamin D for children and adults (as shown in Table 2) (88). In general, normal range of vitamin D is defined by 25(OH)D levels between 30 and 100 ng/ml, which some authors suggest is the appropriate range to ensure the immunomodulatory effects of vitamin D (89). Population studies that found an inverse relationship 25(OH)D and the risk of several types of cancers, cardiovascular diseases, autoimmune diseases and all-cause mortality described reported serum levels of 25(OH)D in the range of 40–60 ng/mL (100–150 nmol/L) (5, 10–12, 60). In order to maintain this range with minimal sunlight exposure, a person would need to consume 4,000–6,000 IUs of vitamin D daily (54). Vitamin D intake recommendations vary between populations; the European Food Safety Authority (EFSA) recommends a daily intake of 600 IU (15 μg) with a maximum limit of 4,000 IU/day (100 μg/day) for healthy adults; while the Institute of Medicine (US) recommends 600 IU/day for children older than 1 year of age and adults below 70 years of age (90). Moreover, when there are no comorbidities, EFSA has reported that doses of ≤ 10,000 IU/day are safe (90). Current guidelines recommend maintenance of circulating levels between 40 ng/mL and 60 ng/mL (100–150 nmol/L) and identify levels <20 ng/mL (50 nmol/L) as vitamin D deficiency and levels between 20–29.9 ng/mL as vitamin D insufficiency (as shown in Table 1) (17).

**Table 2:** Current age-related recommended daily vitamin D supplementation levels [references (17, 86–90)].

**Age**	**Recommended**	**Upper limit**
	**amount**	**recommended**
Birth to 1 year of age	10 μg (400 IU)	25 μg (1000 IU)
Children (1–12 years)	15 μg (600 IU)	60 μg (2400 IU)
Adolescents (12–18 years)	15 μg (600 IU)	75 μg (3000 IU)
Adults (18–17 years)	15 μg (600 IU)	100 μg (4000 IU)
Elderly (>70 years)	20 μg (800 IU)	100 μg (4000 IU)

Vitamin D status is generally determined by the measurement of 25(OH)D concentration in serum rather than by the measurement of the serum biologically active form [1,25(OH)_2_D], as their half-life of these two forms are substantially different, 15 days vs. 15 h, respectively (91). The way on which vitamin D is measured is an important issue, since in the majority of intervention studies mainly focus on vitamin D3. Vitamin D3 supplements effectively elevate plasma levels of 25(OH)D at a rate of 1.5 ± 0.9 nmol/L per supplied 40 IU/day (92). Therefore, to reach serum 25(OH)D levels in the range of 25–125 nmol/L (10–50 ng/mL), requires an intake of about 16–24 nmol/L per day. Since recommended serum levels of 25(OH)D are based on pre-COVID-19 era, studies on the role of vitamin D in SARS-CoV-2 might need modification of the recommended 25(OH)D levels. Recently, it was advised to perform RCT with vitamin D supplementation at a dose that would raise the serum 25(OH)D level above 100 nmol/L (40 ng/ml), arguing that many intervention studies used a too low dose of vitamin D (91).

Presently, regarding the evidence of vitamin D supplementation in COVID-19, the heterogeneity in study design is the biggest hurdle in gathering more conclusive data. Particularly, not having available recent information on the vitamin D status of the participants, variation in clinical end-points as well as different formulations of vitamin D used (e.g. rapid-acting oral calcifediol vs. oral vitamin D3 which is a slower-acting treatment) which result in highly variable outcomes of vitamin D supplementation (88). Well-designed studies including, not restricted to large-scale RCTs are urgently needed to establish the role of vitamin D supplementation in preventing occurrence and severe manifestations of COVID-19 (87).

### Vitamin D Deficiency in the Clinical Progression of SARS-CoV-2

Currently, evidence suggests that being vitamin D-deficient is a risk factor for COVID-19 and that correcting this deficiency may mitigate the risk for clinical progression of COVID-19 (82). Vitamin D may reduce the burden of infection by lowering viral replication rates and by decreasing the pro-inflammatory cytokines and increasing concentrations of anti-inflammatory cytokines (91–93). Additionally, vitamin D may cooperate with type I interferons (IFNs) to control the early phase of SARS-CoV-2 infection (94). Type I IFNs induce strong antiviral activity and there is growing evidence that a weak or delayed type I IFN response contributes to COVID-19 severity and the reduced levels of IFN-α/β could be due to the presence of neutralizing antibodies or due to a reduced type I IFN secretion from plasmacytoid dendritic cells (56, 95). It has been shown that IFN-α and vitamin D display a synergistic inhibition of SARS-CoV-2 replication, whereby vitamin D potentiates the IFN-α action (96). The intensity of the transcriptional activity of IFN-α protein was found to correlate inversely with the severity of the clinical symptoms of COVID-19 (97).

A retrospective case-control study measured 25(OH)D in 216 hospitalized COVID-19 patients and 197 population-based controls and identified lower levels 25(OH)D in hospitalized patients compared to controls, mean ± SD 13.8 ± 7.2 ng/ml compared to 20.9 ± 7.4 ng/ml (*p* < 0.0001) with vitamin D deficiency found in 82.2% of COVID-19 cases and in 42.7% of controls (98). The researchers acknowledge the limitations of this observational study: small sample size and a single-center study. A retrospective study measured 25(OH)D in 74 COVID-19 hospitalized patients and found that the mean serum 25(OH)D concentration was significantly lower in the deceased (13.83 ng/mL ± 12.53 ng/mL) compared to the discharged patients (38.41 ng/mL ± 18.51 ng/mL) (*p* < 0.001); additionally higher levels of 25(OH)D were associated with less extent of total lung involvement (β = 0.10, *p* = 0.004) and levels of 25(OH)D <25 ng/mL were associated with an increased risk of mortality (hazard ratio = 4.15, *p* = 0.04) (99). In the UK, a study among hospital staff showed that vitamin D deficiency or being black, Asian or of ethnic minority descent were independent predictors for seroconversion; with a multivariate analysis showing an odds ratio of 2.6 (95% CI 1.41–4.80; *p* = 0.002) for vitamin D deficiency for SARS-CoV-2 seroconversion, adjusting for age, gender, BMI, ethnicity, comorbidities and job role (100). This finding seems to correlate with a prospective study done in the US that analyzed 5,081 blood samples of black women and found that the ORs for COVID-19 were 1.48 (95% CI 0.95–2.30) for women with 25(OH)D levels of 20–29 ng/mL and 1.69 (95% CI 1.04–2.72) for women with OH(25)D levels <20 ng/mL (p trend 0.02) (101). An Israeli cohort study concluded that low levels of 25(OH)D almost doubled the risk for hospitalization due to COVID-19 (102). Additionally, a meta-analysis showed a strong correlation between death rate caused by SARS-CoV-2 and vitamin D blood levels, reporting that a threshold level of 30 ng/mL decreased mortality considerably (103). A multi-center study measured [25(OH)D_3_] in patients that had acute COVID-19, patients that had healed from COVID-19 and non-infected patients; the study showed that acute COVID-19 patients had the lowest levels of [25(OH)D_3_] (9,63 ± 8.70 ng/mL), followed by healed patients (11.52 ± 4.90 ng/mL, p>0.05), with the highest levels reported by non-infected patients (15.96 ± 5.99 ng/mL, *p* = 0.0091) (104). The most recent systematic review and meta-analysis which involves approximately two million adults suggests that vitamin D insufficiency/deficiency increases susceptibility to COVID-19 and to severe COVID-19, with association regarding mortality reported as less robust (105).

Nonetheless, the role of vitamin D deficiency in the COVID-19 pandemic has limited research mostly restricted to observational studies, which is the main criticism for those who oppose vitamin D supplementation as a public health measure. Nevertheless, one could argue that most studies meet the Bradford Hill criteria that allow, but not prove, to infer causality from associations (106). Observational studies are undoubtedly a lower standard of evidence compared to experimental studies, more prone to bias and confounding variables and should not be used to irrefutably establish causality. However, there is piling evidence of observational studies and a few RCTs that are starting to publish results (discussed in the next section) which could make a difference in scientific approach to the role of vitamin D as a mitigating strategy not only during the pandemic stage but also during the upcoming endemic stage of COVID-19.

## Vitamin D Supplementation During The Covid-19 Pandemic And Beyond

**Figure 3:**
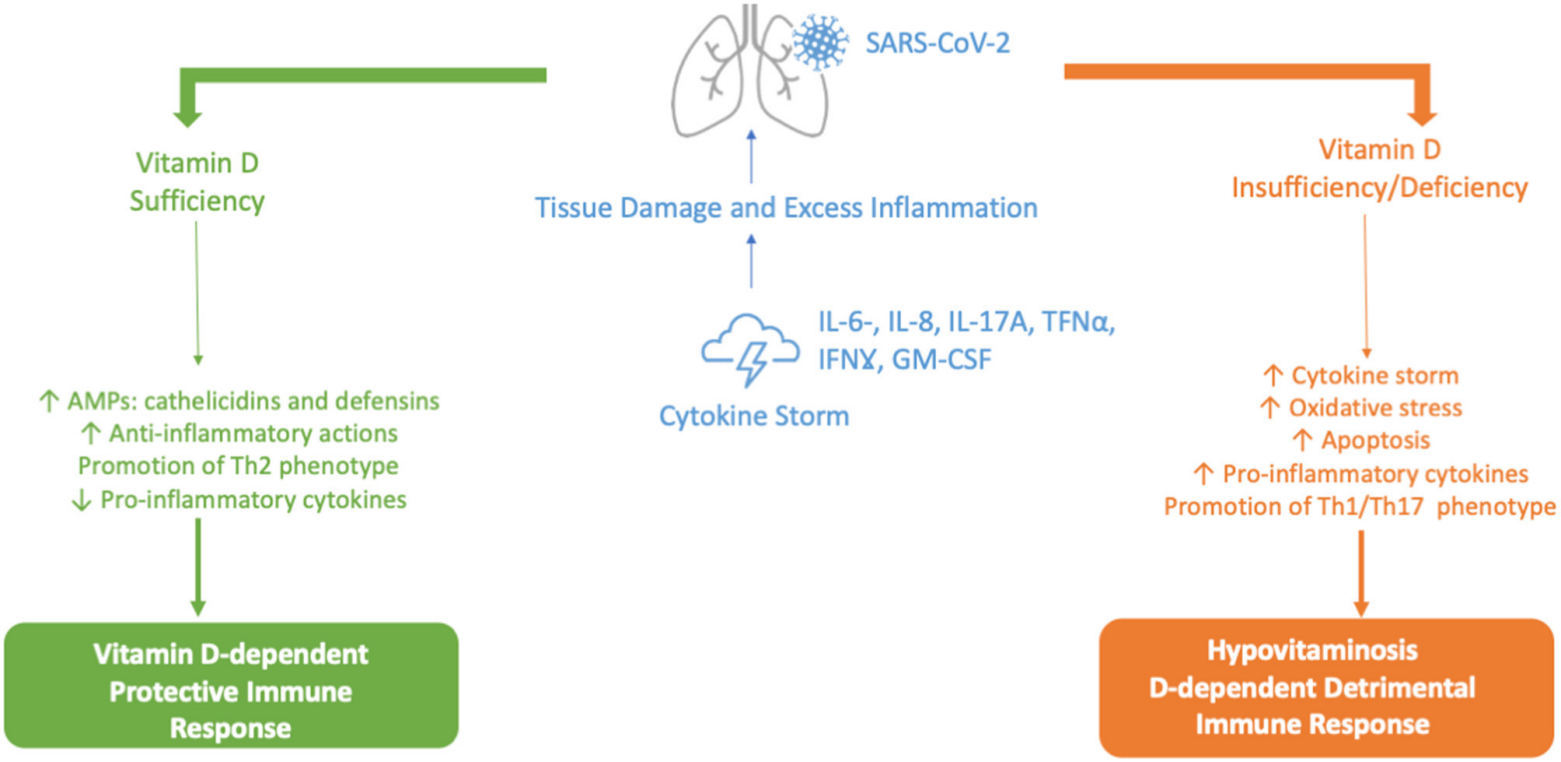
Vitamin D-dependent Protective vs. Hypovitaminosis D-dependent Detrimental Immune Response against SARS-CoV-2. AMPs, antimicrobial peptides; GM-CSF, granulocyte-macrophage colony-stimulating factor; IL-6, IL-8, IL-17A, interleukin 6, 8 and 17A; IFNγ, interferon-gamma; Th1, Th2, Th17, type 1 T helper cells, type 2 T helper cells, type 17 T helper cells; Th; TNFα, tumor necrosis factor alpha.

Meltzer et al. proposed that people who were previously vitamin D deficient but had received treatment did not have an increased risk for COVID-19, compared to those who remained vitamin D deficient; suggesting a protective role of vitamin D supplementation (as shown in Figure 3) (107). Nevertheless, the confidence intervals on estimated rates for these groups were too wide to exclude the possibility of a no-treatment effect. Hastie et al. evaluated the association between vitamin D deficiency and a positive COVID-19 test in the UK Biobank data, reporting no statistically significant association (108). However, the lack of treatment follow-up plus the fact that vitamin D levels were measured between 10 to 14 years before the COVID-19 pandemic are relevant criticisms of this study. Moreover, upon further analysis of the UK Biobank data, it was reported that although circulating vitamin D levels did not affect the risk of COVID-19, the habitual use of vitamin D supplements was associated with a 35% lower risk of COVID-19 (*p* = 0.034) (109).

**Figure 4:**
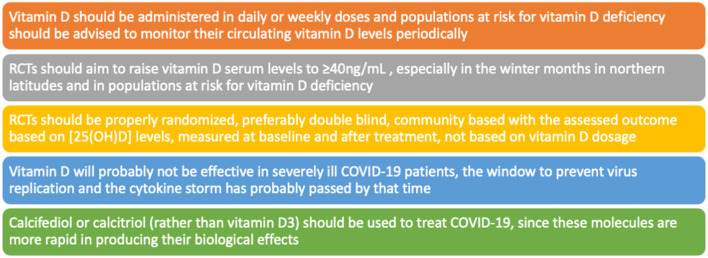
Authors recommendations regarding vitamin D research and supplementation.

A systematic review of RCT found 43 RCTs (48,488 participants) that analyzed vitamin D supplementation for the prevention of acute respiratory infections (ARIs) and reported a small but significant protective effect of vitamin D supplementation on the risk of having one or more ARIs compared with placebo (OR 0.92 [95% CI 0.86–0.99]); adding that the significant heterogeneity amongst the trials may have contributed to an underestimation of a protective effect of vitamin D supplementation (110). Moreover, the authors found vitamin D supplementation to be safe and that protection was associated with daily doses of 400–1000 IU (10–25 μg/day) for up to 12 months, although it is important to point out that direct implication for COVID-19 are not described in this study. An overview of studies dealing with vitamin D supplementation in respiratory tract infections is presented in Supplementary Table 2. As mentioned before, it appears that vitamin D has a greater benefit prior to immune activation during severe illness, which could explain the absence of benefit of vitamin D supplementation in critically ill patients and when doses are administered daily. This concurs with another meta-analysis that concluded that vitamin D supplementation was not only safe but also effective when doses are administered daily (79). This concurs with the findings of Thacher who concluded as well that the most effective way to administer supplementation of vitamin D in order to cause an effect is daily or weekly (111). Therefore, single bolus dosing studies are using a less effective treatment option of administering vitamin D and, in some cases, the short study time does not provide enough time for the administered vitamin D to show sufficient activity in contrast to the rapid progression of SARS-CoV-2, particularly in patients admitted to the intensive care unit (ICU). Additionally, it appears that if vitamin D supplementation begins when COVID-19 patients are admitted to the ICU this might be too late after the onset of symptoms and the window for vitamin D to prevent virus replication as well as the cytokine storm has probably passed (112). Between 4 weeks and up to 3 months supplementation should be provided to significantly increase serum levels of [25(OH)D]; therefore, follow-up time is crucial to prove or disprove an effect of vitamin D supplementation (113, 114). A summary of these recommendations are shown in Figure 4.

Moreover, studies with monoclonal antibodies for COVID-19 reported that the effects of vitamin D status differs between prevention vs. treatment of severe COVID-19; with vitamin D appearing to have greater benefit before immune activation, which again would explain the absence of benefit of vitamin D supplementation in severe cases of COVID-19 (111).

Presently, intervention studies reporting on serum levels of vitamin D provide strong indications for a potential effect of vitamin D supplementation in preventing severe COVID-19 by showing that participants who are initially vitamin D deficient or insufficient are likely to show beneficial effects of vitamin D treatment until normal healthy levels of vitamin D are reached (115, 116) (as shown in Table 3). On the other hand, participants with a healthy vitamin D status are likely not to show any significant beneficial effect of vitamin D supplementation (10). In the largest cohort, dose-response relationships between COVID-19 and serum 25(OH)D measurements in the preceding year were evaluated (116). The risk of SARS-CoV-2 positivity continued to decline until the serum 25(OH)D levels reached 55 ng/ml; an inverse relationship between circulating 25(OH)D levels and SARS-CoV-2 positivity was observed. Several well-designed studies, including but not limited to large scale RCTs, are currently ongoing and will be paramount to define the role of vitamin D supplementation in preventing SARS-CoV-2 infection and/or mitigating the clinical course of COVID-19 (116).

**Table 3:** Summary of interventional studies of vitamin D supplementation associated with COVID-19 outcomes, studies involving single dose vitamin D supplementation have been excluded.

**Study type**	**Study population**	**Aim**	**Results**	**Reference**
Parallel pilot randomized open label, double-masked clinical trial	76 patients hospitalized with COVID-19	Effects of calcifediol treatment	Administration of a high dose of Calcifediol or 25-hydroxyvitamin D, significantly reduced the need for ICU treatment of patients requiring hospitalization due to proven COVID-19	Entrenas et al. (115)
Randomized placebo controlled	40 adults	Effect of high dose, oral cholecalciferol supplementation on SARS-CoV-2 viral clearance; daily 60 000 IU of cholecalciferol (oral nano-liquid droplets) for 7 days	Greater proportion of vitamin D-deficient individuals with SARS-CoV-2 infection turned SARS-CoV-2 RNA negative with a significant decrease in fibrinogen on high-dose cholecalciferol supplementation	Rastogi et al. (117)
Randomized, open label pilot study	50 adults hospitalized with COVID-19	Effect of treatment with calcitriol (0.5 μg/day for 14 days)	A significant reduction in oxygen requirements in patients who received calcitriol	Elamir et al. (31)
Randomized clinical trial	69 RT-PCR SARS-CoV-2 + hospitalized adults mild to moderate disease	Effect of 5,000 IU/day vs. 1,000 IU/day orally for 2 weeks of vitamin D3	5,000 IU daily oral vitamin D3 supplementation for 2 weeks reduces the time to recovery for cough and gustatory sensory loss among patients with sub-optimal vitamin D status and mild to moderate COVID-19 symptoms	Sabico et al. (118)

Pereira et al. published one of the first systematic reviews and meta-analyses that analyzed the association between vitamin D deficiency and COVID-19 severity and found that individuals with severe COVID-19 present 65% more vitamin D deficiency compared to those with mild disease (119). Nonetheless, there are limitations to this meta-analysis, such as the fact that it was based on observational studies, vitamin D dosage strategies were not always clearly reported, most studies chosen presented a high risk of bias, confounding factors were not taken into account in most studies and the data results were not stratified according to the sex of the participants which can influence the results since we know morbidity and mortality of COVID-19 differs for men and women (120, 121). More recently, a systemic review and meta-analysis was published taking into account comorbidities and ethnicity; it also included studies from different geographical locations, the vitamin D cut-off values were clearly defined plus the analysis included a heterogeneity assessment and the authors tested for publication bias as well (122). They reported that most COVID-19 patients were suffering from vitamin D deficiency or insufficiency plus that vitamin D deficient patients had three times a higher chance of SARS-CoV-2 and five times a higher probability of developing severe disease (122). Once again the authors note that for more reliable findings, larger clinical trials are needed.

Additional studies have assessed interleukin-6 (IL-6) levels in COVID-19 patients in relation to vitamin D, since it has been theorized that low levels of serum [25(OH)D] increase IL-6 synthesis (123). In a small group of patients with COVID-19, chronic kidney disease and vitamin D deficiency, the researchers observed a steep and significant decrease in circulating IL-6 levels when correcting the vitamin D deficiency (104). Ling et al. reported that cholecalciferol booster therapy was associated with a reduced risk of COVID-19 mortality (adjusted OR 0.13, 95% CI 0.05–0.35, *p* < 0.001) (124). In a RCT, 40 subjects were given 60,000 IU of cholecalciferol, oral nano-liquid droplets per day for 7 days, and found that a larger proportion of vitamin D deficient individuals that received vitamin D supplementation became negative (SARS-COV-2 RNA levels) before day 21 when compared to the placebo group (63 vs. 21%); additionally, the fibrinogen levels on the high dose supplementation group decreased (117).

Even though the COVID-19 pandemic has put vitamin D supplementation in the spotlight, it has also made it very difficult for high quality RCTs studies to be explored. The severe and urgent nature of the pandemic favors quicker results with an abundance of observational studies conducted in COVID-19 patients. Moreover, the COVID-19 patient population can be quite heterogenous and there are several methodological limitations for experimental studies regarding vitamin D supplementation such as optimal dosage level, comorbidities in the sample population and lack of standardization in determining deficiency levels (125). A properly designed RCT should be done in the community, because correcting vitamin D deficiency in patients that are already sick enough to be hospitalized with COVID-19 may be too late. Participants would need to be recruited once identified as vitamin D deficient and then receive either supplementation or placebo (91). Moreover, the assessed outcome should be based on 25(OH)D levels, measured at baseline and after treatment, not based on vitamin D dosage as it happens in most studies. According to established guidelines for a correctly designed RCT to measure vitamin D-related outcomes, serum 25(OH)D is defined as the accepted indicator (126). Recommendations for vitamin D supplementation are summarized in Figure 4.

Thus, even though current evidence suggests that vitamin D intake may be useful, effective and safe as part of the protocol in the treatment of COVID-19 patients; to date we still lack enough data to state this categorically, as the majority of studies involved a small number of patients (82). Currently there are 21 ongoing studies by the Cochrane Library as of interest to assess whether vitamin D supplementation is an effective and safe treatment for COVID-19 (127).

## The Future Of Vitamin D Beyond The Covid-19 Pandemic

The role of vitamin D is now known to be wide and involved in several biological processes and the vitamin D endocrine system has been linked to several common diseases including cancer, cardiovascular disease, osteoarthritis, tuberculosis, autoimmune disorders and neuropathies (128–131). Several reasons are listed for low vitamin D status: (1) seasonal lack of UVB radiation, (2) decreased outdoor activities, (3) the growing aging population and (4) low proportion of vitamin D rich food in the diet (91).

Currently, the common belief in the scientific community is that SARS-CoV-2 will become endemic and will continue to cause outbreaks in regions where it appeared to have been eliminated (2, 3, 132). Therefore, governments should continue to evaluate evidence regarding mitigating strategies that are quick, deployable, cost effective and readily available to fight COVID-19. Among these strategies, we propose that vitamin D supplementation in those who have a vitamin D deficiency or insufficiency can have a role to support protective immune response to SARS-CoV-2, particularly for what concerns the innate immune response, which is not sensitive to COVID-19 variants. Previous experimental and clinical data have shown that vitamin D deficiency increases the risk of ARIs, increases the risk of asthma and exacerbates chronic obstructive pulmonary disease (COPD) (68, 79). Therefore, correcting vitamin D deficiency would improve the overall respiratory health of the population as SARS-CoV-2 becomes endemic and joins other viral respiratory illness such as influenza and respiratory syncytial virus (RSV) (1–3).

Ideally, individuals at risk for vitamin D deficiency should adopt practices that can naturally improve their vitamin D levels such as regular exposure to direct sunlight and addition to their diet of food sources rich in vitamin D. The second is possible but not always attainable and the first is a challenge in the northern hemisphere where sunlight is already limited because of the climate plus the necessary sun exposure can be considered risky by many and therefore is avoided (133). This explains why the third option, vitamin D supplementation, is the most viable way to increase vitamin D levels (134). Vitamin D supplementation is considered to be safe and poses little risk to the health of individuals (1, 91, 117, 135, 136); with special care taken particularly among the elderly when it is used in combination with other supplements such as calcium or magnesium (137). Moreover, the European Society for Clinical Nutrition and Metabolism (ESPEN) recommends that individuals at risk or infected with COVID-19 correct their vitamin D deficiency via oral supplementation (138).

From a research point of view, the need for intervention trials is imperative to assess the effect of vitamin D supplementation on COVID-19 risk and disease related outcomes with a homogeneity in study design and dose regimen (135, 139, 140). During the COVID-19 pandemic, a few governments have taken an open and transparent initiative addressing vitamin D deficiency as a public health problem, by evaluating all the available evidence by a group of experts and putting forward recommendations for the development of nationwide policies (141, 142). Based on the current evidence, it would be advisable for more governments to take a similar approach. Moreover, governments as well as public health institutions will need to lead the way as we enter the endemic stage of SARS-CoV-2; therefore, funding properly designed RCTs that produce results that will settle the issue in the near future is imperative. If the benefits of correcting vitamin D deficiency in the general population are confirmed, the improvement of the overall respiratory health of the population will have an impact not only on SARS-CoV-2 infection but also on other seasonal viruses that are responsible for hospitalizations and deaths every year (143). If proper RCTs are not performed the controversy surrounding vitamin D supplementation, which was an issue before the COVID-19 pandemic, will continue and the possible health benefits of vitamin D supplementation will remain in perpetual scientific limbo.

For now, the clinical evidence that we have indicates that vitamin D supplementation may reduce susceptibility to viral infections (79, 144). A reasonable and safe tolerable upper intake level (UL) should be 10,000 IU vitamin D/day, which corresponds to a serum 25(OH)D concentration of approximately 100 ng/mL (17). The individual genetic profile has a strong influence on vitamin D associated metabolic pathways with an impact on immune regulation with single nucleotide polymorphisms (SNPs) from a number of genes involved in the vitamin D metabolism associated with lower or higher circulating vitamin D levels or affecting the vitamin D receptor (VDR)-driven functional consequences of vitamin D (69). Therefore, unraveling how genetics influences serum 25(OH)D levels is important for identifying persons at risk of vitamin D deficiency and improving understanding of the observed association between vitamin D deficiency and several diseases, including COVID-19 (68).

**Figure 5:**
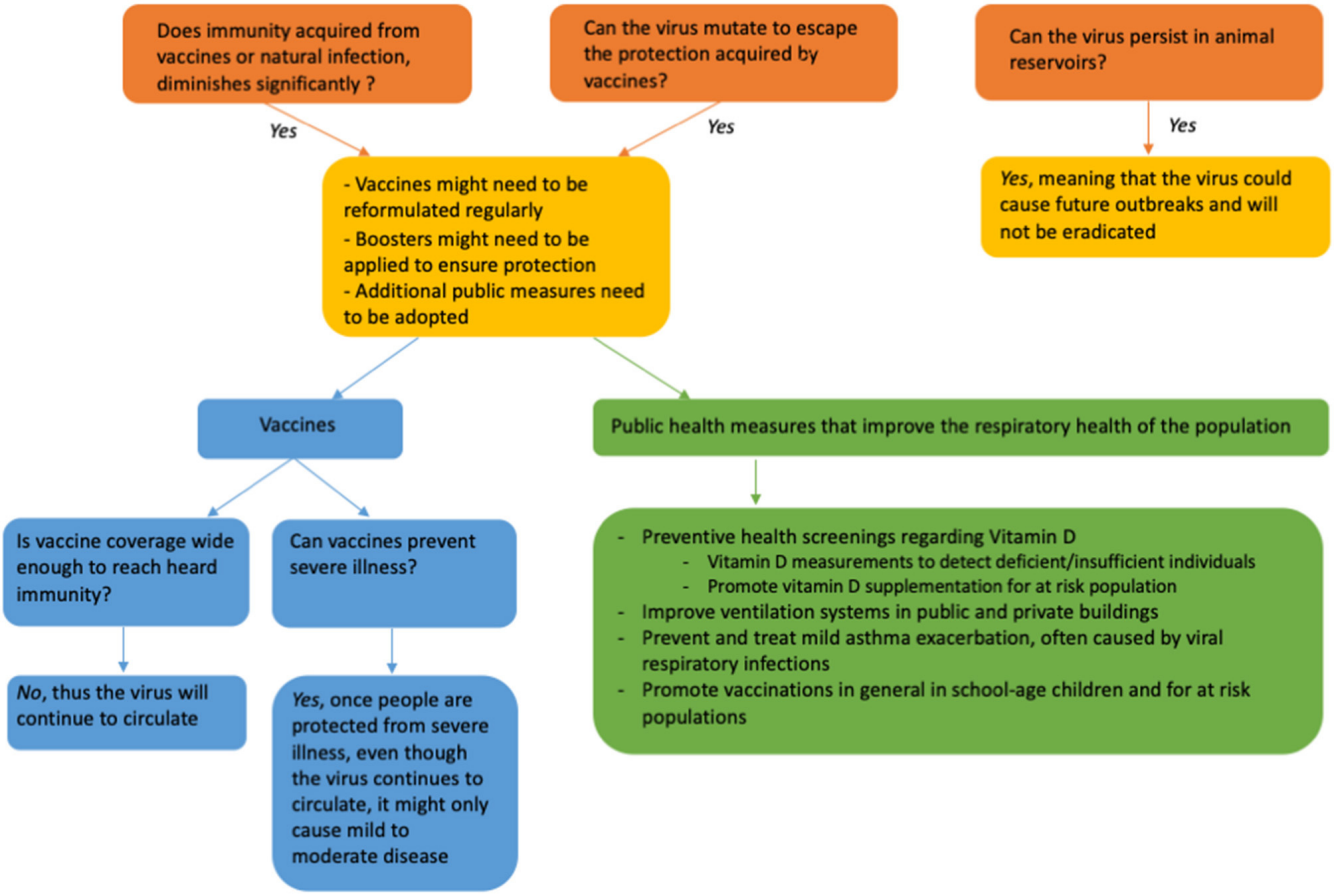
Forecasting the future for the COVID-19 pandemic, including the possible role for vitamin D supplementation.

The majority of the scientific community sees a future where COVID-19 becomes endemic; thus, we need to learn to co-exist with SARS-CoV-2 (3). To be clear, this does not mean to have public health measures that ameliorate the impact of endemic COVID-19 are not necessary, quite the opposite; if anything, the current pandemic has shown all the pitfalls in our current health care systems and if we want to be better prepared to handle the next pandemic, we need to fix those problems but also deploy preventive measures that improve the overall health status of our population. Together with the vaccination campaigns, vitamin D supplementation is a low cost, easy to administer and widely available intervention (as shown in Figure 5) (111, 145).

## Conclusion

In summary, the immunomodulatory role of vitamin D is involved in the production of AMPs, promoting autophagy and increasing degrading enzymes in macrophages plus suppressing the development of the “cytokine storm”, by increasing IL-10 producing regulatory T cells, while inhibiting Th17 cells and promoting the production of virus-specific IgG1 antibodies. Moreover, vitamin D affects the expression of the ACE_2_ as the main binding partner of the viral spike-protein and inhibits the renin-angiotensin system (RAS).

**Figure 6:**
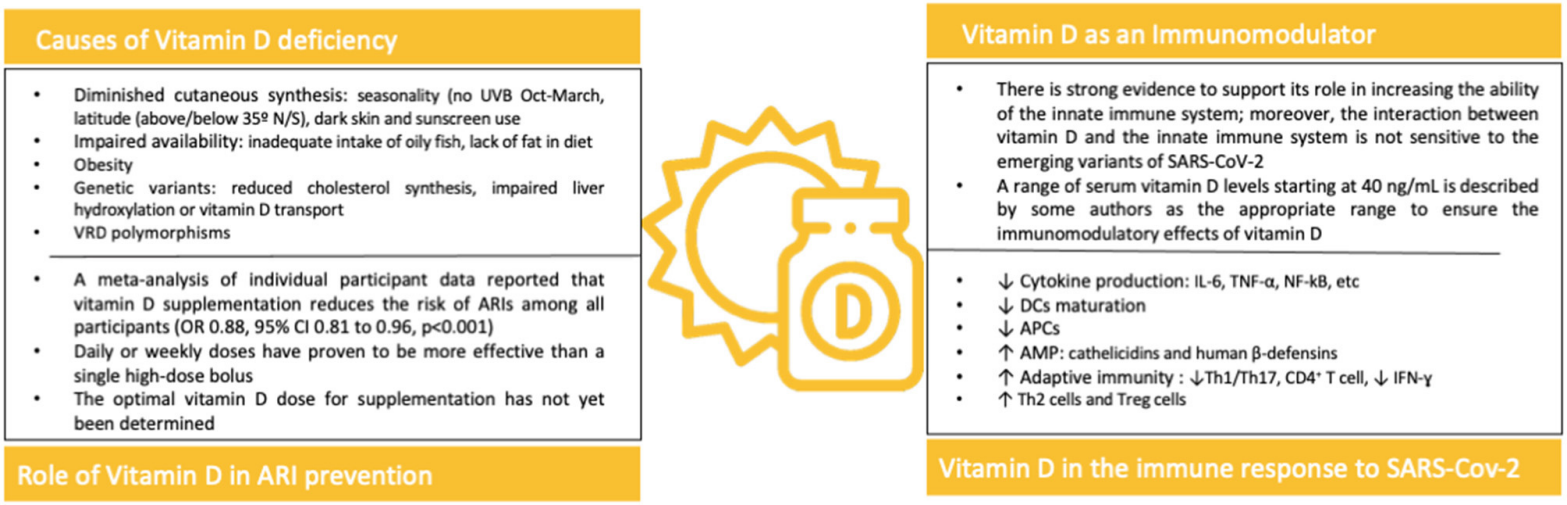
Summary of key review points.

There is no doubt that conclusive evidence from properly designed large RCTs is still required about the role of vitamin D in COVID-19. Nonetheless, in COVID-19 the existing immune dysregulation associated with an increased inflammatory condition could be corrected properly by the immunomodulatory activity of vitamin D. Moreover, vitamin D supplementation, especially in vitamin D deficient or insufficient individuals, can reduce the development of severe symptoms in a preventative strategy up to the development of mild symptoms of COVID-19. It is however crucial to properly provide vitamin D supplementation, which entails repeated administration over sufficient amount of time, preferably in a preventive setting with sufficient follow up time to observe a clinical effect. Moreover, evidence shows that vitamin D is not only safe but is provides protection against acute respiratory tract infections overall, which will contribute to the overall respiratory health of the population when SARS-CoV-2 becomes endemic as it is predicted to occur in the near future. A summary of the key points expressed in this review are shown in Figure 6.

Regarding vitamin D dose it has previously been reported that every 4 ng/mL increase in circulating 25(OH)D levels a 7% decreased risk of seasonal respiratory infection was found (146). Similar numbers have been reported for COVID-19, with Kaufman et al. suggesting 1.6% lower risk of SARS-CoV-2 positivity per ng/mL (135). Therefore, it is a reasonable approach to provide vitamin D supplementation of 800 to 2,000 IU (20 to 50 μg) daily to populations at risk for vitamin D deficiency and to the general population living in the northern hemisphere during the winter months in order to prevent vitamin D deficiency as a public health measure (147). This daily dose maintains 25(OH)D concentrations in the range of 30 to 40 ng/mL in healthy adults and has shown no harm when administered, as summarized in the Supplementary Table 2 (148–154).

## Author Contributions

DB and HS: conceptualization, writing, and editing. All authors have read and agreed to the final version of the manuscript.

## Conflict of Interest

The authors declare that the research was conducted in the absence of any commercial or financial relationships that could be construed as a potential conflict of interest.

## Publisher's Note

All claims expressed in this article are solely those of the authors and do not necessarily represent those of their affiliated organizations, or those of the publisher, the editors and the reviewers. Any product that may be evaluated in this article, or claim that may be made by its manufacturer, is not guaranteed or endorsed by the publisher.
