# Supplementary material for: Vitamin D: A Potential Mitigation Tool for the Endemic Stage of the COVID-19 Pandemic?
Source: Front Public Health. 2022 Jun 10;10:888168. doi: 10.3389/fpubh.2022.888168 (PMC9226430; doi:10.3389/fpubh.2022.888168)
Supplement: Supplementary file 1 [file Table_1.docx]

**Supplemental Table 1.** Studies on the levels of vitamin D and increased risk of acute lower respiratory tract infection (ARLI) in childhood and upper respiratory tract infections (URTI) in adults.

| **Study Type** | **Study Population** | **Aim** | **Results** | **Reference** |
| --- | --- | --- | --- | --- |
| Hospital-based case control | 150 children, 80 cases and 70 controls, age 2 to 60 months | Association of serum 25-hydroxyvitamin D3 with severe ALRI, controlling for demographic and other potential risk factors | Vitamin D levels >22.5 nmol/L associated with lower risk of severe ALRI | Wayse V., et al [24] |
| Comparative study | 105 children <5 years old with ALRI vs healthy controls | Association between vitamin D deficiency and respiratory infection by comparing serum [25(OH)D] levels | Significantly lower vitamin D levels in children admitted to PICU | McNally J.D., et al [25] |
| Case control study | 64 children aged 1-25 months with ARLI vs healthy controls | Association between vitamin D status with the risk of ALRI | Similar vitamin D concentrations among cases and controls | Roth D.E., et al [26] |
| Case control study | 25 children aged 1 to 18 months with ARLI vs 25 healthy controls | Association between vitamin D status and ALRI | Significantly lower vitamin D in ARLI than in controls | Roth D.E., et al [27] |
| Multicentre prospective cohort study | >1000 children hospitalized with bronchiolitis | Association between circulating [25(OH)D] status at admission and disease severity among infants hospitalized for bronchiolitis | Vitamin D deficiency correlates to increased risk of intensive care admission and longer hospital stay | Vo P., et al [28] |
| Observational study | 18,883 participants, > 12 years old | Association between [25(OH)D] level and recent URTI; adjusting for demographics and clinical factors | [25(OH)D] levels were inversely associated with recent URTI; with stronger association in those with respiratory tract diseases. | Ginde A.A., et al [29] |
| Cross-sectional study | 6789 participants aged 45 to 47 | Association between the current vitamin D status, [25(OH)D], with respiratory infections and lung function | Each 4ng/ml increase of [25(OH)D] associated with 7% lower risk of respiratory infection (95%, CI 3, 11%) | Berry D.J., et al [30] |
| Cross-sectional study | 2000 non—institutionalized adults aged 65 and older | Association between serum [25(OH)D] concentrations and respiratory diseases | Increased risk of respiratory diseases (including influenza and pneumonia) when [25(OH)D] below 20 nmol/L | Hirani V. [31] |

ARLI: acute lower respiratory tract infection; URTI: upper respiratory tract infection.

**Supplemental Table 2.** Vitamin D supplementation for treatment of childhood and adult respiratory tract infections (studies involving single dose vitamin D supplementation have been excluded)

| **Study Type** | **Study Population** | **Dose and Duration of Supplementation** | **Results** | **Reference** |
| --- | --- | --- | --- | --- |
| Randomized, double-blind, placebo control study | 89 infants with bronchiolitis | 100 IU/kg/day for at least 5 days during hospital stay | Significant improvement in the duration of hospitalization and time taken to improve oral feeding. | Saad et al [148] |
| Randomized trial | Over 300 school-age children | 1200 IU/day during winter months | Reduced influenza A infections | Urashima et al [149] |
| Randomised controlled superiority trial | Over 3000 children | 100,000 IU once every 3 months for 18 months | No decrease in incidence of pneumonia | Manaseki-Holland et al [150] |
| Randomized trial | 247 children | Milk fortified with vitamin D from January to March | Significantly lower ARI episodes during the study period | Camargo et al [151] |
| Randomized controlled trial | 1330 healthy children and adolescents | 14,000 IU/week for 8 months | Similar incidence of influenza but moderately reduced incidence of other respiratory viral infections | Loeb et al [152] |
| Randomized controlled trial | 162 adults | 2000 IU/day of vitamin D3 during 12 weeks of winter | No difference in incidence, severity or duration of URTI. Most subjects had sufficient vitamin D levels at the start | Li-Ng et al [153] |
| Randomized controlled trial | 140 adults | 4000 IU/day as oil drops for one year | Respiratory tract infections reduced by 23% in vitamin D when compared to placebo. | Bergamn et al [154] |

ARI: acute respiratory infections; IU: international unit; URTI: upper respiratory tract infection
