# Supplementary figures and images for: Vitamin D: A Potential Mitigation Tool for the Endemic Stage of the COVID-19 Pandemic?
Source: Front Public Health. 2022 Jun 10;10:888168. doi: 10.3389/fpubh.2022.888168 (PMC9226430; doi:10.3389/fpubh.2022.888168)

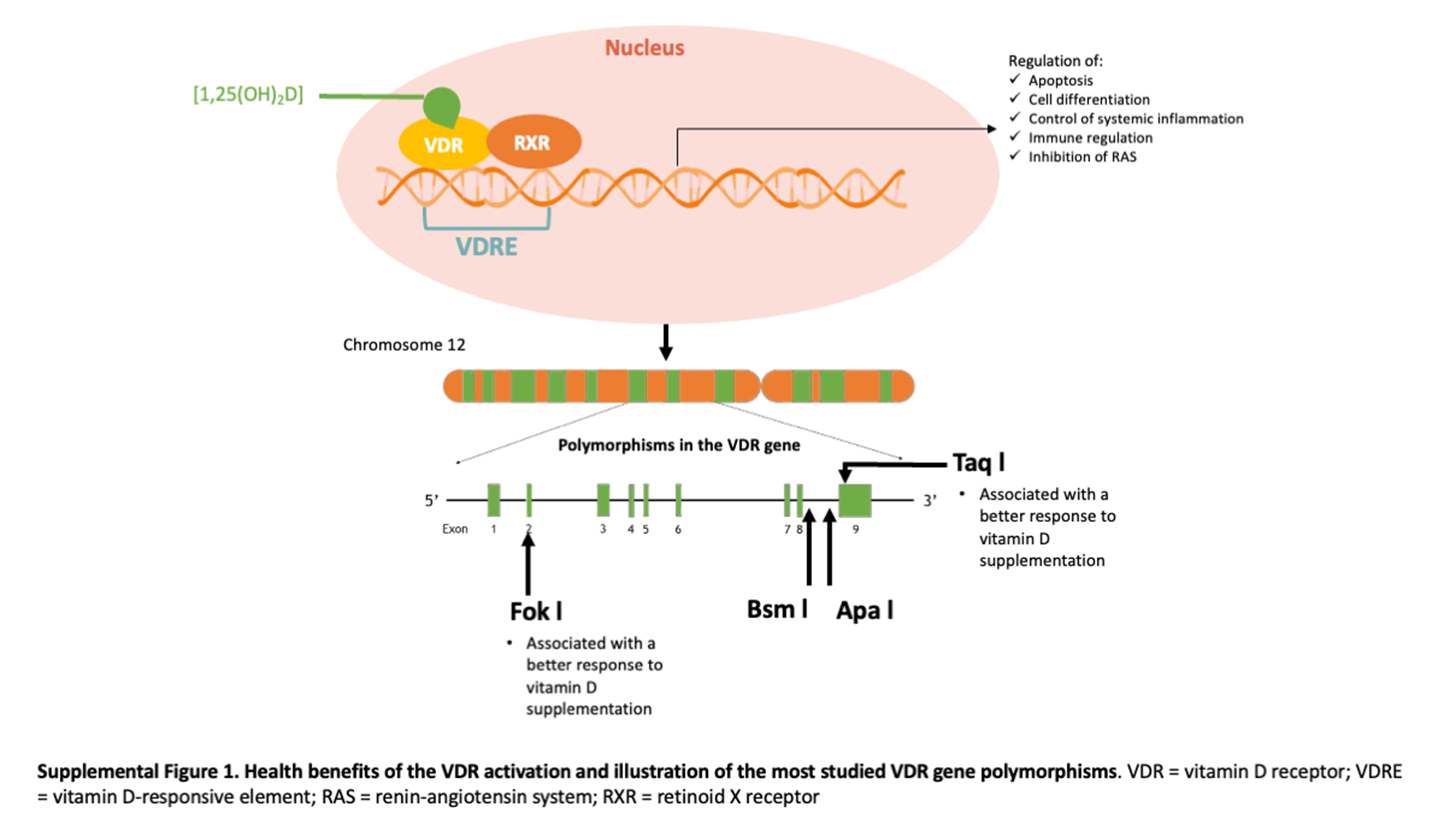

Supplement: Supplementary file 2 [file Image_1.tiff]
